# Enzymatic Poly(gallic acid)‐grafted *L*‐Histidine Reduces Cell Inflammation and Oxidative Stress: In Silico and In Vitro Studies

**DOI:** 10.1002/cbdv.71266

**Published:** 2026-04-27

**Authors:** Carmen G. Hernández‐Valencia, Iris N. Serratos, Carlos M. Torre‐Morales, Rosa Isela Ortiz‐Huidobro, Alfredo Vázquez, José Pedraza‐Chaverri, Valentín Martínez‑López, Javier Fernández‑Torres, Roberto Sánchez‑Sánchez, Yessica Zamudio‑Cuevas, Miquel Gimeno

**Affiliations:** ^1^ Facultad de Química, Departamento de Alimentos y Biotecnología Universidad Nacional Autónoma de México, Ciudad Universitaria Ciudad de México México; ^2^ Departamento de Biotecnología Universidad Autónoma Metropolitana‐Iztapalapa Iztapalapa, Ciudad de México México; ^3^ Departamento de Química Universidad Autónoma Metropolitana‐Iztapalapa Iztapalapa, Ciudad de México Mexico; ^4^ Facultad de Química, Departamento de Química Orgánica Universidad Nacional Autónoma de México, Ciudad Universitaria Ciudad de México México; ^5^ Facultad de Química, Departamento de Biología Universidad Nacional Autónoma de México, Ciudad Universitaria Ciudad de México México; ^6^ Unidad de Ingeniería de Tejidos Terapia Celular y Medicina Regenerativa Instituto Nacional de Rehabilitación “Luis Guillermo Ibarra Ibarra Ciudad de México México; ^7^ Laboratorio de Liquido sinovial, Instituto Nacional de Rehabilitación Luis Guillermo Ibarra Ibarra Ciudad de México México

**Keywords:** computational model, electrostatic interactions, l‐histidine, oxidative stress, poly(gallic acid), proinflammatory cytokines

## Abstract

Enzyme‐mediated poly(gallic acid) (PGAL) exhibits intrinsic anti‐inflammatory and antioxidant properties; in this study, its chemical functionality was enhanced by microwave‐assisted grafting of L‐histidine to obtain PGAL‐His. Incorporation of the amino acid into the PGAL backbone reached 33.24 ± 0.74 mol%. Molecular docking and binding energy calculations were used to model PGAL‐His interactions, revealing that the NH_3_^+^ and COO^−^ groups, together with the nitrogen atoms of the imidazole ring, play a central role in stabilizing electrostatic interactions. In vitro assays demonstrated reduced adhesion of inflammation‐associated immune cells in a THP‐1 macrophage model. Furthermore, PGAL‐His significantly decreased reactive oxygen species (ROS) levels and reduced the secretion of the pro‐inflammatory cytokines IL‐6, TNF‐α, and IL‐1β in THP‐1 cell cultures. These results indicate that PGAL‐His is a promising chemically modified polyphenolic material with potential applications as a therapeutic tool for the management of immune‐mediated inflammatory diseases.

## Graphical abstract

Microwave‐assisted grafting yields a PGAL–His conjugate with enhanced binding through electrostatic and hydrogen‐bond interactions. The material modulates THP‐1 macrophage response, reducing ROS production, pro‐inflammatory cytokines (IL‐6, IL‐1β, TNF‐α), and cell adhesion. These findings highlight its potential as a therapeutic biomaterial for inflammatory diseases

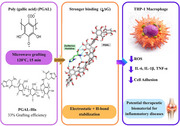

## 1 Introduction

Therapeutic strategies for immune‐mediated inflammatory diseases, most notably rheumatoid arthritis (RA) and Crohn's disease, commonly focus on alleviating inflammation and pain to slow disease progression, primarily using conventional nonsteroidal anti‐inflammatory drugs (NSAIDs), disease‐modifying antirheumatic drugs (DMARDs), and corticosteroids [1, 2, 3]. However, these therapies are associated with limited long‐term efficacy and substantial off‐target toxicity affecting multiple organs [4]. Recent in vitro studies have revealed that naturally derived polyphenolic compounds can help reduce inflammatory processes and among them, the poly(gallic acid) (PGAL), enzymatically obtained from gallic acid (GA), exhibits a stable helical structure and remarkable antioxidant capacity owing to its multiradical stability, characteristics that enable the reduction of proinflammatory cytokines and reactive oxygen species (ROS) [5, 6, 7, 8, 9].

Previous studies have shown that the properties of PGAL can be enhanced through nontoxic microwave‐assisted grafting of quaternary amine–containing amino acids, conferring antimicrobial activity against pathogenic bacteria [10, 11]. Therefore, the functionalization of PGAL to enhance its biological properties motivated us to investigate its grafting with *L*‐histidine (His), an essential amino acid that plays a crucial role in cell regeneration and tissue repair [12]. The ionizable groups of His at different pKa values of 1.8, 6.0, and 9.2 have been studied for molecular docking and binding energy calculations considering two protonation states; the less predominant protonated form with a positively charged imidazole ring and the predominantly neutral zwitterionic form at physiological pH, in which the 1,3‐azole moiety remains neutral [13]. This heterocyclic and aromatic amino acid has biological properties such as a proton buffering capacity, the ability to chelate metal ions, and antioxidant activity. Regarding the latter characteristic, various studies have shown that his has potential as a therapeutic agent for inflammatory conditions, including NSAID‐induced gastric erosion, which is commonly associated with RA, Crohn's disease, ulcerative colitis or gout treatments [14]. In patients with RA for instance, a decrease in His concentration is related to protein‐energy depletion, inflammation episodes and oxidative stress in cells [15]. In the present study, microwave‐mediated grafting of *L*‐His onto PGAL (PGAL–His) resulted in reduced levels of the pro‐inflammatory cytokines IL‐1β, IL‐6, and TNF‐α, as well as decreased cell adhesion and intracellular ROS generation in a THP‐1 cell model. This macrophage‐like, differentiable human monocytic leukemia cell line enables a detailed evaluation of cytokine production in a context that mimics innate immune–driven inflammatory environments, thereby constituting a well‐established in vitro model for studying inflammatory responses implicated in diseases such as RA, inflammatory bowel disease, sepsis, and atherosclerosis. Moreover, it provides a reliable platform for investigating inflammation‐related mechanisms relevant to other conditions, including psoriasis, systemic lupus erythematosus, and cancer‐associated inflammation, thus facilitating the assessment of inflammatory modulation and the identification of potential therapeutic strategies using this novel compound. Despite the well‐documented antioxidant and anti‐inflammatory properties of PGAL, its biological performance can still be limited by the lack of functional groups that enhance specific molecular interactions and cellular responses. Although amino acid grafting has been shown to improve certain properties of PGAL [10, 11], the potential of L‐His given its unique imidazole functionality, buffering capacity, and known role in inflammation modulation remains insufficiently explored in this context. Therefore, this work is undertaken to design and characterize a novel PGAL–His material obtained via microwave‐assisted grafting, and to elucidate its molecular interactions through in silico approaches alongside its biological effects in vitro. Specifically, we aim to evaluate whether His functionalization enhances the anti‐inflammatory and antioxidant properties of PGAL by assessing cytokine production, cell adhesion, and ROS generation in a THP‐1 macrophage model, thereby providing insight into its potential as a therapeutic strategy for immune‐mediated inflammatory diseases.

## 2 Materials and Methods

### 2.1 Materials

Lacasse from *Trametes versicolor* (LTV, lyophilized protein stored at 5°C; ≥10 U/mg) was purchased to Merck (USA) and used as received. GA, ABTS sodium phosphate (mono and dibasic), 2,2‐diphenyl‐1‐picrylhydrazyl (DPPH), 2,4,6‐tripyridyl‐striazine (TPTZ), 2,20‐azobis‐(2‐methylpropionamidine) dihydrochloride (AAPH), fluorescein, 6‐hydroxy‐2,5,7,8‐tetramethylchroman‐2‐carboxylic acid (trolox), ethylenediaminetetraacetic acid (EDTA), N,N´‐dimethylthiourea (DMTU), and paraformaldehyde (PFA), methanol, ethanol, crystal violet were purchased from Merck (USA). Trypan blue, and Phorbol 12‐myristate 13‐acetate (PMA) were from Merck. Flaked sodium hydroxide was provided by CONQUIMEX (Mexico). Roswell Park Memorial Institute Medium (RPMI‐1640/F12) complete medium, Hank's medium, 0.25% trypsin‐EDTA dissociation reagent, phosphate‐buffered saline (PBS) pH 7.2, penicillin, and streptomycin (P/S) and were supplied by Gibco (USA). Fetal bovine serum (FBS) was provided by ByProducts (USA). Histidine was supplied by FagaLab (Mexico). DCFDA / H2DCFDA—Cellular ROS Assay Kit was from Abcam (Ab113851).

### 2.2 Synthesis of PGAL and PGAL‐His

Enzymatic PGAL was synthesized from GA according to the method described by Lopez et al. [5]. For grafting, PGAL (100 mg), L‐histidine (92.1 mg), and zinc sulfate monohydrate (20 mg) were dissolved in 6 mL of 100 mM phosphate buffer (pH 7) in a 25 mL round‐bottom flask equipped with a magnetic stirrer. The mixture was placed in a Discover SP CEM microwave reactor (USA) and heated at 120°C for 15 min. The reaction mixture was then poured into cold ethanol (1:10 v/v), dried, and reconstituted in water. Purification was achieved by centrifugation using 3 kDa shear filtration units (Merck, USA). The resulting supernatant was lyophilized to obtain PGAL and PGAL–His as a black powder.

### 2.3 Characterization of PGAL and PGAL‐His

Number‐average molecular weight (*M*_n_) and polydispersity index (PDI) of the samples were determined by size exclusion chromatography (SEC) using an Agilent HPLC system (USA) equipped with a refractive index detector. Two ultra‐hydrogel 500 columns (7.8 × 300 mm, Waters, USA) were connected in series and maintained at 30°C. The system was calibrated with polyethylene glycol standards. Samples were eluted with ultrapure deionized water (Simplicity UV, Millipore, USA) containing 0.1 M LiCl at a flow rate of 0.6 mL/min. All samples were dissolved in the mobile phase and filtered through 0.45 µm membranes prior to injection. Infrared (IR) spectra were recorded on an ATR‐FTIR spectrometer (Perkin Elmer, Spectrum 100) over the range of 250–4000 cm^−^^1^. To quantify the incorporated amino acid, 20 mg of polymer was dissolved in 2.5 mL of 2 M NaOH and stirred at 60°C for 24 h. The mixture was then precipitated with 10 mL of ethanol at 5°C. The supernatant was collected, titrated, and diluted. Aliquots of 500 µL were mixed with 500 µL of 0.001% TNBSA solution, shaken, and incubated at 37°C for 2 h. The reaction was stopped with 125 µL of 1 N HCl, and absorbance was measured at 335 nm. Amino acid concentration was determined using a calibration curve prepared with *L*‐histidine.

### 2.4 Antioxidant Activity Measurements

Stock aqueous solutions of PGAL (2 mM) and PGAL–His (2 mM) were prepared for in vitro antioxidant assays. The 2,2‐diphenyl‐1‐picrylhydrazyl radical (DPPH•) scavenging activity was determined spectrophotometrically by mixing 100 µL of sample with 100 µL of freshly prepared 80 µM DPPH• solution. The mixtures were vortexed and incubated in the dark for 30 min before measuring absorbance at 517 nm. Ferric reducing antioxidant power (FRAP) was assessed using a reagent mixture containing 1.7 mM FeCl_3_ and 0.8 mM TPTZ in 300 mM acetate buffer (pH 3.6). In a 96‐well microplate, 30 µL of sample solution was combined with 300 µL of the FRAP reagent. After standing for 15 min, absorbance was measured at 583 nm using a Biotek Synergy HT spectrofluorometer (Winooski, VT, USA). Antioxidant capacity was calculated against a Trolox calibration curve (0–500 µM). Oxygen radical absorbance capacity (ORAC) was determined in 75 mM phosphate buffer (pH 7.4). In each well of a 96‐well microplate, 25 µL of sample, 25 µL of 153 mM AAPH, and 150 µL of 4 nM fluorescein solution were combined. Fluorescence was monitored every minute for 90 min at 37°C using excitation and emission wavelengths of 485 nm and 525 nm, respectively. Hydroxyl radical (HO•) scavenging activity was evaluated using terephthalate as a probe, which forms fluorescent hydroxyterephthalate upon hydroxylation. HO• radicals were generated via a Fenton reaction using 180 µL of a mixture containing 0.2 mM ascorbic acid, 0.2 mM FeCl_3_, 0.2 mM EDTA, 1 mM H_2_O_2_, and 1.4 mM terephthalate in 50 mM phosphate buffer (pH 7.4). This mixture was combined with 20 µL of sample or 20 µL of distilled water as a control. HO• generation proceeded at room temperature for 30 min, and FeCl_3_ was pre‐mixed with EDTA before addition. Fluorescent adducts were measured at 326 nm (excitation) and 432 nm (emission) using the Synergy HT spectrofluorometer. Data from DPPH• and ORAC assays were analyzed using Probit statistical analysis in the NCSS program to determine IC_50_ values (half‐maximal inhibitory concentration).

### 2.5 Molecular Docking Studies

*PGAL*: A fully protonated nine‐unit oligomer of PGAL was constructed and optimized at the M06‐2X/6‐311++G(d,p) level of theory. This structural model was developed by our research group [8, 16]. His: We constructed histidine using the program Avogadro in two states. For this, we considered two protonation states of histidine, the protonated and zwitterionic at physiological pH. We carried out molecular docking studies considering the following systems: PGAL‐histidine (Figure 2a) and PGAL‐histidine (Figure 2b). Those systems were docked in USFC Chimera by triplicated each one, obtaining more than 50 different complexes each in pdb format. Besides, those files were prepared adding charge and radii of the atoms in a pqr file to determinate the Δ*G*_b_.

### 2.6 Electrostatic and Non‐electrostatic Contributions to Δ*G*_b_

We determined both electrostatic and non‐electrostatic contributions to evaluate the Δ*G*_b_ of PGAL with the two histidine species (protonated and zwitterionic), following the approach described described by Baker et al. [17].

(1) $$\Delta G_{b}=\Delta G_{\text{elec}}+\Delta G_{\text{non}-\text{elec}}$$

The electrostatic contributions to binding can be further divided into two main components, providing a more detailed understanding of the interaction forces involved:

(2) $$\Delta G_{\mathrm{elec}}=\Delta G_{\mathrm{solv}}+\Delta G_{\mathrm{coul}}$$

The solvation energy (Δ*G*_solv_) and Coulombic energy (Δ*G*_Coul_) were calculated for both the complexes and the free species using the Adaptive Poisson–Boltzmann Solver [17].

For electrostatic calculations, atomic charges, atomic radii and dielectric constants were required. During the docking assays, atomic charges for PGAL and histidine were assigned according to the force field parameters implemented in AutoDock Vina [18], using structures previously prepared with UCSF Chimera. Atomic radii were assigned using the PDB2PQR web server (https://server.poissonboltzmann.org/pdb2pqr). Dielectric constants of 78 for water [19] and 3.0 for PGAL [20] were used; the latter value was selected based on structural similarities to polymers such as polyimide and polypropylene, which typically exhibit dielectric constants in the range of 2.0–4.0. The value of 3.0 is consistent with reported dielectric properties of polyimides and related polymers [21]. These electrostatic components were computed at physiological pH.

Non‐electrostatic energy (Δ*G*_non‐elec_) was estimated by multiplying the change in solvent accessible surface area (ΔSASA) upon complex formation by an interfacial tension coefficient (γ = 5 cal•mol^−1^ Å^−2^) [22, 23], following standard approaches to quantify hydrophobic contributions.

(3) $$\Delta G_{\mathrm{non}-\mathrm{elec}}=\gamma \left(ASAP_{\mathrm{PGAL}-\mathrm{His}}-ASA_{\mathrm{PGAL}}-ASA_{\mathrm{His}}\right)$$

The solvent‐accessible surface area (ASA) of both the complexes and the free species was calculated using the visual molecular dynamics (VMD) program [24], applying a standard probe radius of 1.4 Å. For each system, the pose exhibiting the most favorable Δ*G*_b_ was chosen for detailed analysis of the molecular interactions.

### 2.7 Culture Conditions for THP‐1 Macrophages

The human monocytic leukemia (clone 1) (THP‐1) cell line (ATCC TIB‐202 RRID: CVCL_0006) was maintained in RPMI‐1640 medium supplemented with 10% heat‐inactivated fetal bovine serum (FBS) and 1% penicillin/streptomycin (P/S). Cells were incubated at 37°C with 5% CO_2_ (Heracell 150i, ThermoFisher Scientific), and the medium was replaced every three days. For experiments, cells were seeded in T25 flasks at a density of 2.5 × 10^5^ cells/mL and grown to confluence (∼1 × 10^6^ cells/mL). Differentiation into adherent macrophage‐like cells was induced by treatment with 100 ng/mL phorbol 12‐myristate 13‐acetate (PMA) for 3 h. Following differentiation, cells were washed to remove PMA, centrifuged, and the pellet was resuspended in fresh RPMI‐1640 medium without PMA. Cells were then transferred to T75 flasks containing medium supplemented with 10% FBS and incubated overnight. Untreated THP‐1 cells were included as controls.

### 2.8 Cytokine Quantifications in THP‐1 Activated Cells

Non‐activated and PMA‐activated THP‐1 cells were seeded at 1 × 10^4^ cells per well in 48‐well plates containing RPMI‐1640 medium supplemented with 2% FBS and 1% P/S. Cells were incubated for 24 h at 37°C in 5% CO_2_ and 95% humidity (Heracell 150i, ThermoFisher Scientific) to allow adherence. After attachment, the medium was removed, and treatments were applied for 24 h and 48 h. Culture supernatants were collected and stored at −20°C until analysis. Levels of IL‐1β, IL‐6, and TNF‐α were quantified using enzyme‐linked immunosorbent assays (ELISA) according to the manufacturer's instructions (PeproTech Human Standard ABTS ELISA Development Kit, Catalogue #900‐K95, 900‐K16, and 900‐K25, respectively). Absorbance was measured in 96‐well plates using a BioRad iMark plate reader.

### 2.9 Cell Adhesion by Crystal Violet Assay in THP‐1 Cell Line

Non‐activated and PMA‐activated THP‐1 cells were fixed with 4% paraformaldehyde (PFA) at 4°C. After removing the PFA, cells were washed with distilled water and stained with 0.5% crystal violet for 10 min. Excess stain was removed, and cells were rinsed several times with distilled water. Stained cells were observed using an Axiovert microscope (Zeiss, USA). Finally, methanol was added to each well, and absorbance was measured at 575 nm using a BioRad iMark microplate reader to compare treatments.

### 2.10 Oxidative Stress Determination in THP‐1 Cell Line

Oxidative stress in activated and non‐activated THP‐1 cells was evaluated after 24 and 48 h of exposure to the test materials. Intracellular ROS were measured using 25 µM carboxy‐H_2_DCFDA, a fluorogenic ROS indicator, in calcium/magnesium‐supplemented PBS (Ca/Mg PBS). Cells were incubated with the dye for 30 min at 37°C in a 5% CO_2_ atmosphere with 95% humidity. Following incubation, the dye was removed, and cells were washed three times with Ca/Mg PBS by centrifugation at 11,000 rpm for 5 min at 25°C (Eppendorf 5424R). ROS levels were quantified by flow cytometry (FACSCalibur, BD Biosciences). Geometric means of carboxy‐DCF fluorescence distributions, measured at excitation/emission wavelengths of 495/529 nm, were calculated using CellQuest Pro software, version 5.2.1 (BD, San Jose, CA, USA).

### 2.11 Statistical Analysis

All experiments were performed in quadruplicate and repeated in at least three independent experiments. Data were analyzed using GraphPad Prism version 8. Two‐way analysis of variance (ANOVA) was applied for balanced data, followed by Tukey‐Kramer multiple comparisons of means. Differences were considered statistically significant at *p* ≤ 0.05.

## 3 Results and Discussion

### 3.1 Characterization and Antioxidant Activity for PGAL‐His

**FIGURE 1:**
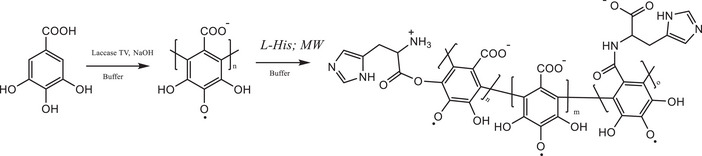
Proposed reaction scheme for the synthesis of PGAL‐His from GA.

The reaction scheme for the grafting of the amino acid onto PGAL chain is shown in Figure 1, together with the proposed molecular structure for the final product. This procedure is relevant because microwave‐assisted reactions, in contrast to conventional chemical methods for amide or ester bond formation, proceed under mild conditions and yield products with low cytotoxicity [10, 11, 25].

The SEC measurements (Table 1) show no significant differences in *M*_n_ between PGAL and PGAL‐His, however, there is an increase in polydispersity for the grafted sample, which indicates successful insertion. The molar percentage of insertion determined by TNBSA consumption is 33.24 ± 0.74 mol%, which is higher than those previously reported for *L*‐arginine and *L*‐lysine grafting onto PGAL [10, 11]. The FTIR spectra for *L*‐His and PGAL are shown in Supporting Data 1, where PGAL displays the expected bands, as reported elsewhere [5], whereas new bands at 1154, 617, and 522 cm^−1^ are assigned to PGAL‐His grafting, and signal superposition provides evidence of insertion.

**TABLE 1:** Results for *M*_n_, degree of His incorporation, and antioxidant assays for PGAL and PGAL‐His.

Sample	Grafting (%)	Mn (Da)	PDI	DPPH (IC_50_ mg/ mL)	FRAP (eq. µM trolox)	ORAC (IC_50_ µM/g)	HO• (eq. µM Trolox)
PGAL	—	5561	1.13	0.165 ± 0.007^a^	74.54 ± 3.59^b^	659.2 ± 32.3^b^	10.53 ± 0.27
PGAL‐His	33.24 ± 0.74	5490	1.34	0.263 ± 0.002^b^	53.71 ± 3.33^a^	300.2 ± 24.2^a^	12.13 ± 2.11

*Different letters in the same column mean significant differences (p ≤ 0.05) by Tukey's method.

On the other hand, PGAL scavenges free radicals via single‐electron transfer (SET) mechanism, as reported elsewhere [26], which could have a significant impact on ROS attenuation, and differs from the hydrogen atom transfer mechanism commonly reported for polyphenols [8]. Importantly, the IC_50_ for radical scavenging of DPPH in PGAL‐His increases with respect to PGAL [10, 11], but the activity in the FRAP assay decreases with respect to the parent polymer, a phenomenon also observed in PGAL‐g‐L‐Lys and PGAL‐g‐L‐Arg, which is attributed to the pendant amino acid units that limit electron transfer between the metal center and the polymer backbone [10, 11]. Interestingly, the elimination of hydroxyl radicals (HO·) has no significant differences in the grafted samples, but for the absorption of oxygen radicals by ORAC assay. PGAL‐His reduces the inhibition by approximately half compared to PGAL alone. This process is related to the number of amino groups involved in the scavenging of oxygen radicals generated by the AAPH [11]. Furthermore, the preservation of approximately 33.2% antioxidant capacity in PGAL‐His can be attributed to the antioxidant properties of the imidazole ring [12].

### 3.2 PGAL‐His Complexes: Molecular Docking Studies and Binding Energy Calculations

**FIGURE 2:**
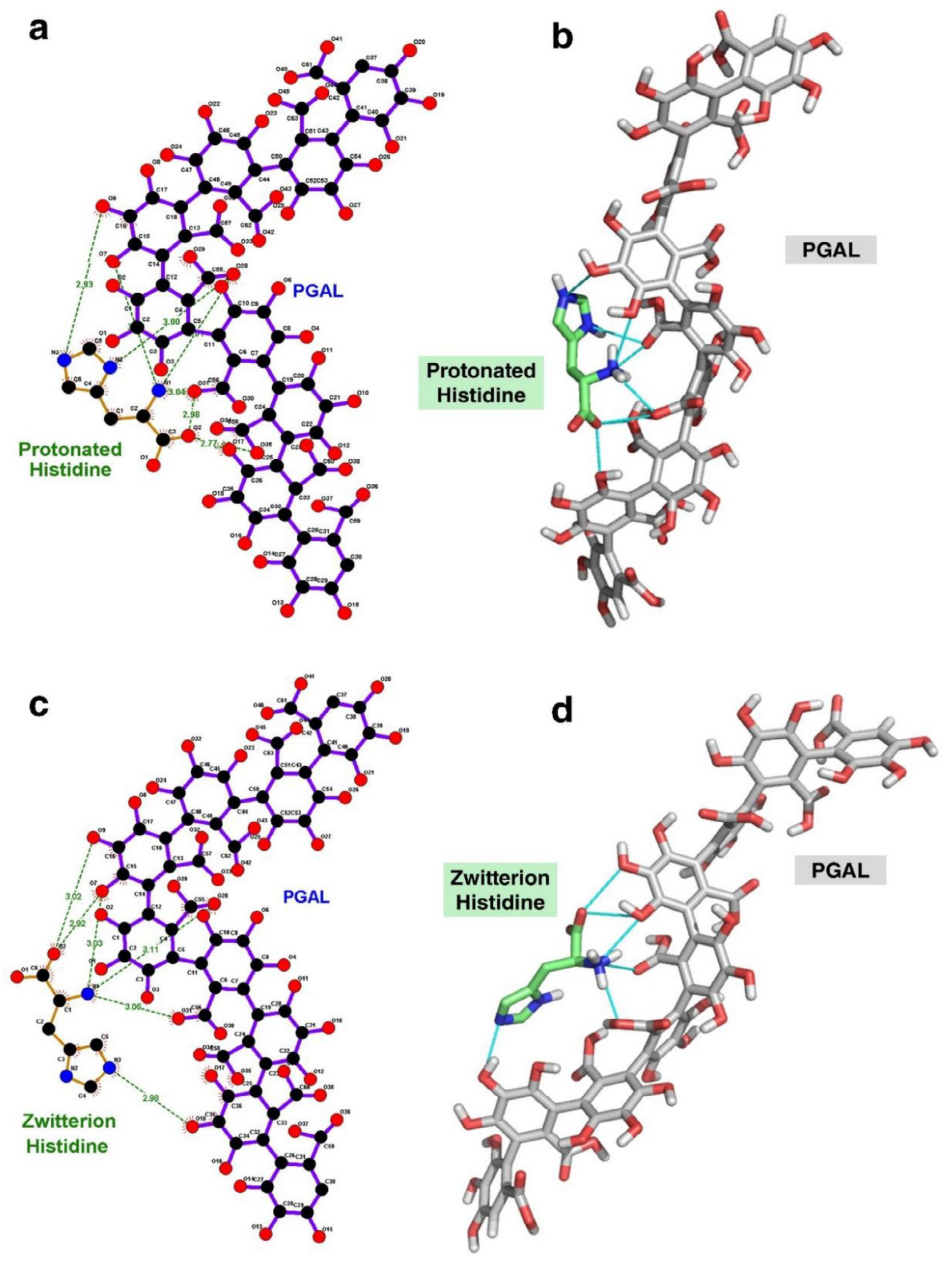
Interaction maps of best complexes obtained by molecular docking assays based on the most favorable Δ*G*_b_ values: PGAL‐protonated His complex (a and b) and PGAL‐zwitterionic His complex (c and d). Green lines indicate hydrogen bonds. Images were generated using LigPlot+ [27] and PyMOL [28] programs.

The molecular docking studies between PGAL and His are conducted considering two protonation states: the less predominant fully protonated form with a positively charged imidazole ring, and the predominantly neutral zwitterionic form at physiological pH. All dockings are evaluated best on the most favorable Δ*G*_b_, and Figure 2 shows the best complex for PGAL‐protonated His and PGAL‐zwitterionic His.

The PGAL‐protonated His complex (Figure 2a, b) establishes at least six hydrogen bonds during binding. This suggests that the NH_3_^+^ group and the nitrogen atoms of the imidazole ring of His form hydrogen bonds with the oxygen atoms of PGAL (NH─O), while the COO^−^ group of His interacts with the hydroxyl (OH) groups of the polymer. However, the PGAL–zwitterionic His complexes (Figure 2c, d) also form six hydrogen bonds, involving similar interactions. Notably, one hydrogen bond involving the imidazole ring is lost, likely due to the absence of a positive charge on the ring in the zwitterionic form.

### 3.3 Δ*G*_b_ Calculations

To better understand the energetically favorable contributions to ΔG_b_ between PGAL and the two protonation states of His, we analyze the electrostatics and non‐electrostatics components shown in Table 2.

**TABLE 2:** Δ*G*_b_ values for the interaction of PGAL‐His were at physiological pH by APBS [17] and VMD1.9.1 [24].

Complexes	Δ*G*_solv_ (kJ/mol)	Δ*G*_Coul_ (kJ/mol)	Δ*G*_non‐elec_ (kJ/mol)	Δ*G*_b_^[+]^ (kJ/mol)
PGAL‐Protonated His	46	−96	−5.0	−55
PGAL‐Zwitterionic His	33	−116	−5.0	−88

$$\left[+\right]\Delta G_{b}=\Delta G_{\mathrm{solv}}+\Delta G_{\mathrm{Coul}}+\Delta G_{\mathrm{non}-\mathrm{elec}}$$

The interpretation of the binding energy for the PGAL‐His system is as follows:

- PGAL‐Protonated His complexes: ΔG_b_ ranges from −25 to −55 kJ/mol.
- PGAL‐Zwitterionic His complexes: ΔG_b_ ranges from −37 to −88 kJ/mol.

These results indicate that the zwitterionic form of His binds slightly more strongly to PGAL, as evidenced by the more negative Δ*G*_b_ values. In both protonated and zwitterionic complexes, the major contribution to binding is electrostatic in nature, specifically Coulombic (charge‐based) interactions. Non‐electrostatic interactions, such as van der Waals forces, also play a role, albeit to a lesser extent. In addition, hydrogen bonds form in both complexes, further stabilizing the interactions (Figure 2). This analysis highlights the critical role of protonation state in modulating PGAL‐histidine interactions and confirms that electrostatics, complemented by specific hydrogen bonding, largely govern the binding affinity.

### 3.4 Anti‐Inflammatory Effect of PGAL‐His in Monocyte/Macrophage THP‐1 Cell Model

**FIGURE 3:**
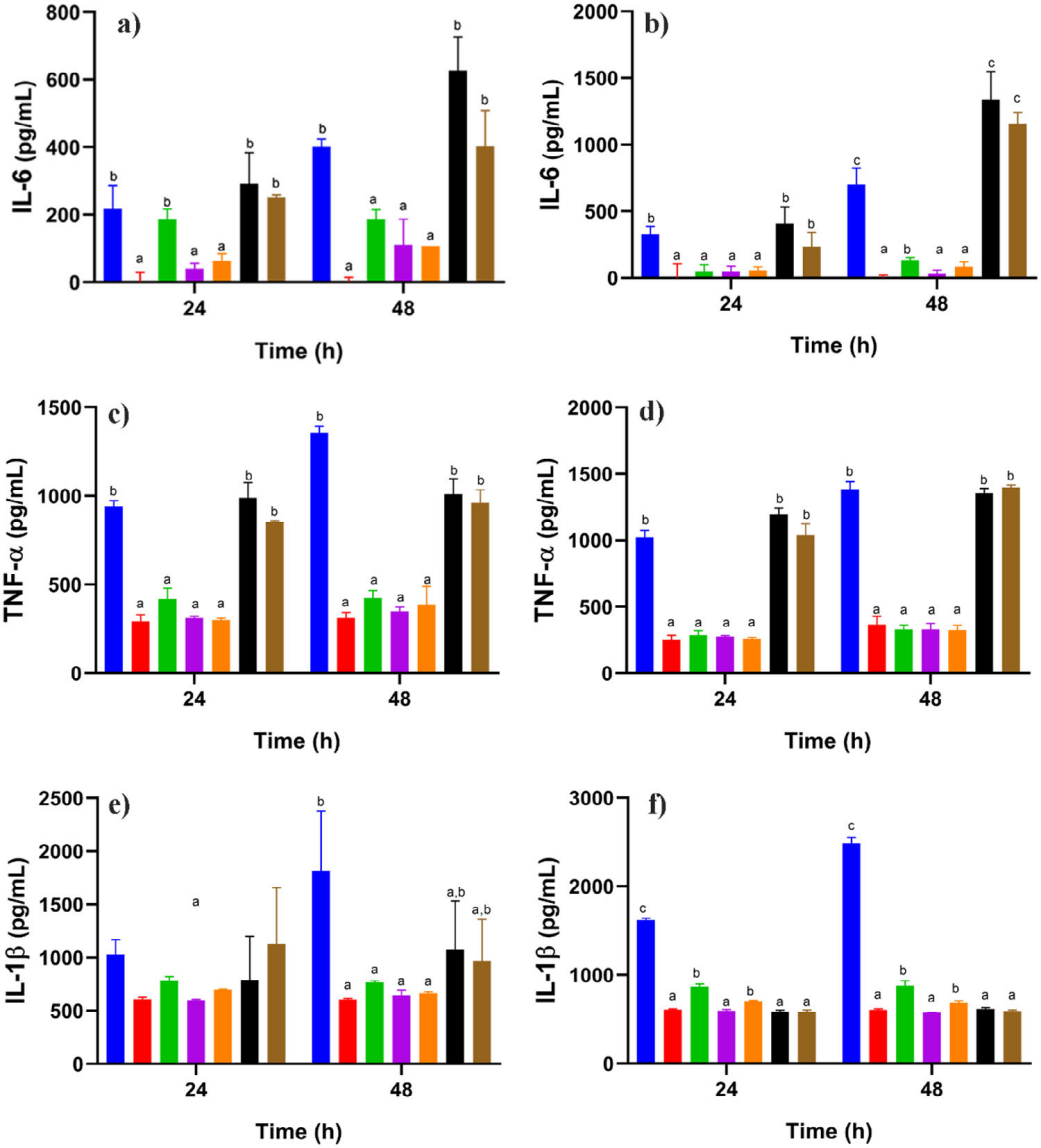
Quantification of IL‐6, TNF‐α, and IL‐1β by ELISA assays. THP‐1 incubated for 24 and 48 h, control (blue) and in the presence of PGAL at 100 µg/mL (red), and 200 µg/mL (green), PGAL‐His at 133 µg/mL (purple), and 266 µg/mL (orange) and L‐His at 33 µg/mL (black), and 66 µg/mL (brown). Quantification of cytokines from non‐activated (panels a, c, e) and activated by PMA (panels b, d, f). Different letters in each graph indicate significant differences established by Tukey's method.

Macrophages play a key role in the pathogenesis of immune‐mediated inflammatory diseases since they are activated by various cytokines and contribute to chronic inflammation and joint damage, which is a characteristic phenomenon. Regulating the balance of macrophage polarization is essential to reduce the inflammatory response and promote disease remission [29, 30]. In this regard, THP‐1 cells provide a suitable model to study the dynamics of inflammatory regulation due to its ability to differentiate into macrophages in the presence of PMA (activating factor) [9]. During differentiation, THP‐1 cells acquire key morphological and functional characteristics, including increased adhesion, a stellate morphology, and elevated production of pro‐inflammatory cytokines such as TNF‐α, IL‐1β, and IL‐6 [31, 32]. In the present study, two concentrations of PGAL, 100 and 200 ppm [9], as well as two concentrations of PGAL‐His (133 and 266 ppm) to maintain identical base PGAL content, are evaluated. Figure 3 shows that PGAL and PGAL‐His reduce the concentration of proinflammatory cytokines after 48 h of exposure for non‐activated THP‐1 and macrophage‐like cells. This effect is attributed to the presence of PGAL, as previously reported by Zamudio‐Cuevas et al. [9].

On the other hand, Hasegawa et al. reported that *L*‐His at a concentration of 20 mM reduced TNF‐α levels [33]. In contrast, we found that an approximately 47‐fold lower concentration of this amino acid than that used in the earlier study resulted in increased levels of IL‐6 and TNF‐α.

### 3.5 Anti‐Adhesion effect of PGAL‐His on THP‐1 Macrophages

**FIGURE 4:**
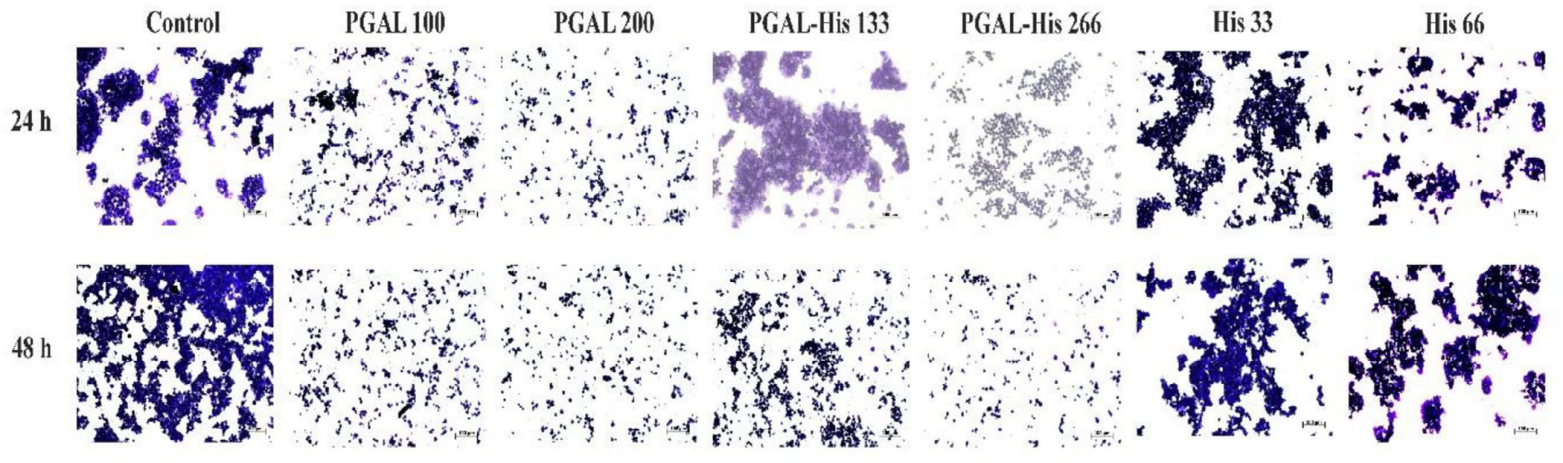
Micrographs for the THP‐1 cells exposed to PGAL, PGAL‐His, His, and control for 24 and 48 h by crystal violet assay. Scale bars correspond to 100 µm. Images obtained with a 40X objective using optical microscopy are representative of one of three independent experiments.

Cell adhesion in the immune response facilitates the extravasation of leukocytes, which is an important step for immune cells to reach the site of inflammation [9]. Cell adhesion of THP‐1 cells under basal conditions and after activation with PMA is significantly increased compared to non‐stimulated cells. The cell adhesion effect induced by PMA is reversed with PGAL in synoviocytes as reported by Zamudio‐Cuevas et al. [34], and this is also observed with PGAL‐His treatment at both concentrations, in contrast to the effect of the amino acid alone (Figure 4).

### 3.6 PGAL‐His Reduces the Production of ROS in THP‐1 Macrophages

**FIGURE 5:**
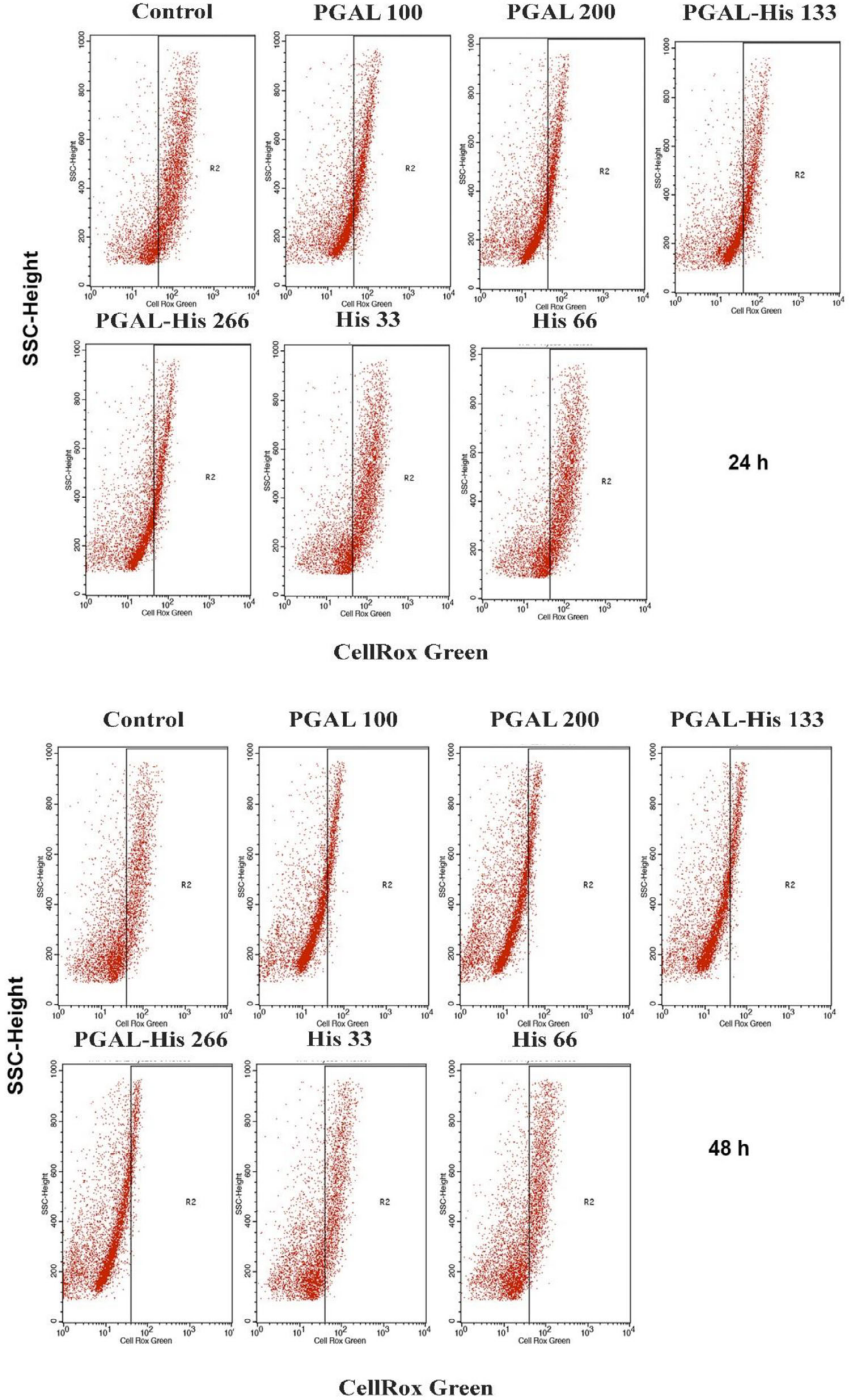
Quantification of ROS in THP‐1 macrophages exposed to PGAL, PGAL‐His, His and control. Representative images of cells analyzed by flow cell cytometry after 24 and 48 h of exposure to the treatments.

In addition to approaches to inhibit inflammation, such as blocking cell adhesion, decreasing cytokine release or activating the immune response, inhibition of ROS production is also being considered [35]. Antioxidant activity assays indicate that PGAL alone scavenge radicals via the SET mechanism [36], however, PGAL‐His exhibits a reduced antioxidant effect. For ROS determination in the THP‐1 macrophage model the control group has the highest percentage of positive cells at both 24 h (62.1%) and 48 h (31.17%), confirming the elevated basal ROS production under PMA stimulation [37, 38]. In contrast, PGAL treatments markedly reduce intracellular ROS detection in a dose‐and time‐dependent manner. At 24 h, PGAL 100 and PGAL 200 µg/mL decrease the positive cells to 33.4% and 22.82%, respectively, corresponding to reductions of 46% and 63% compared to control. This effect is further accentuated at 48 h, where values decrease to 9.03% (PGAL 100 µg/mL) and 4.81% (PGAL 200 µg/mL), representing an inhibition greater than 70%. Similarly, the PGAL‐His also exhibits a notable antioxidant effect, following a dose‐dependent pattern. At 24 h, PGAL‐His133 and PGAL‐His266 reduced ROS‐positive cells to 33.32% and 26.31% (46% and 58% inhibition compared to control). At 48 h, these percentages further decreased to 8.23% and 6.07%, respectively, indicating reductions also greater than 70%. These antioxidant behaviors for PGAL and PGAL‐His are consistent with other reports for polyphenolic compounds, such as GA, which have been shown to modulate oxidative stress in macrophages [39]. Similarly, resveratrol has been reported to reduce ROS levels in *in vitro* models using U1 and THP‐1 macrophages [40]. However, the inhibition achieved in our study is notable, reaching > 70% at 48 h, suggesting that the polymeric structure may provide a more sustained antioxidant response compared to small molecule polyphenols, which are often limited by fast metabolism and physicochemical instability [8, 41]. Other studies have reported that polymerized phenolic compounds exhibit enhanced antioxidant activity and improved stability in aqueous environments compared with their monomeric counterparts [41]. In contrast, treatments with amino acid alone (His33 and His66) show little modulation of ROS levels, as values remain high (57.51% and 59.99% at 24 h, and 29.33% and 29.18% at 48 h), without relevant differences to control. This highlights that the antioxidant capacity observed is mainly associated with the PGAL/PGAL‐His structure which may enhance stability, radical scavenging capacity, or cellular uptake. Similar findings have been reported for conjugated polyphenols, where structural modifications improved bioavailability [42]. Taken together, these findings demonstrate that PGAL and PGAL–His exert a marked and sustained inhibition of ROS production in THP‐1 derived macrophages in a dose‐ and time‐dependent manner, whereas histidine alone shows no significant effect (Table 3 and Figure 5).

**TABLE 3:** Quantification of ROS in THP‐1 macrophages exposed to PGAL, PGAL‐His, His and controls.

Treatments in activated THP‐1	% CellRox Green positive cells at 24 h	% CellRox Green positive cells at 48 h
Control	62.1	31.17
PGAL 100	33.4	9.03
PGAL 200	22.82	4.81
PGAL‐His 133	33.32	8.23
PGAL‐His 266	26.31	6.07
His 33	57.51	29.33
His 66	59.99	29.18

These experimental results suggest that sustained activity of these nontoxic, naturally derived compounds could be advantageous for therapeutic applications in diseases characterized by chronic oxidative stress [43, 44].

## 4 Conclusion

The in vitro results exhibit that microwave‐mediated grafting of L‐His onto the PGAL chain reduces oxidative stress and inflammation in cells. PGAL‐His reduces adhesion of immune cells responsible for inflammatory processes as well as proinflammatory cytokines IL‐1β, IL‐6, TNF‐α, and ROS. These findings suggest that PGAL‐His has great potential as a therapeutic tool in the treatment of chronic inflammatory diseases. Additionally, the computational study displays that the zwitterionic form of His has specific binding to PGAL. This interaction is supported by a favorable binding energy and is primarily stabilized by electrostatic interactions, particularly Coulombic forces, as well as hydrogen bonds, with non‐electrostatic interactions (such as van der Waals forces) contributing to a lesser extent. These findings suggest that even small molecules like histidine can engage in significant interactions with larger macromolecules, owing to the presence of complementary charge distributions or suitable functional groups.

## Author Contributions

**Iris N. Serratos**: investigation, writing – original draft, formal analysis, methodology, software. **José Pedraza‐chaverri**: investigation, funding acquisition, supervision. **Yessica Zamudio‑cuevas**: investigation, funding acquisition, writing – review and editing, supervision, resources. **Valentín Martínez‑lópez**: investigation, supervision, methodology. **Miquel Gimeno**: conceptualization, investigation, funding acquisition, writing – review and editing, project administration, supervision, resources. **Javier Fernández‑torres**: investigation, funding acquisition, supervision. **Alfredo Vázquez**: funding acquisition, supervision. **Rosa Isela Ortiz‐huidobro**: investigation, methodology, data curation. **Roberto Sánchez‑sánchez**: investigation, funding acquisition, supervision. **Carlos M. Torre‐morales**: investigation, methodology, data curation. **Carmen G. Hernández‐valencia**: investigation, writing – original draft, formal analysis, data curation, methodology.

## Conflicts of Interest

The authors declare no conflicts of interest.

## Supporting information




**Supporting File**: cbdv71266‐sup‐0001‐SuppMat.docx

## Data Availability

The data that support the findings of this study are available from the corresponding author upon reasonable request.
