# Supplementary material for: Enzymatic Poly(gallic acid)‐grafted L‐Histidine Reduces Cell Inflammation and Oxidative Stress: In Silico and In Vitro Studies
Source: Chem Biodivers. 2026 Apr 27;23:e71266. doi: 10.1002/cbdv.71266 (PMC13112469; doi:10.1002/cbdv.71266)
Supplement: Supplementary file 1 — Supporting File: cbdv71266‐sup‐0001‐SuppMat.docx [file CBDV-23-e71266-s001.docx]

Supplementary data Supplementary data 1. FTIR for L-His, PGAL and PGAL-His


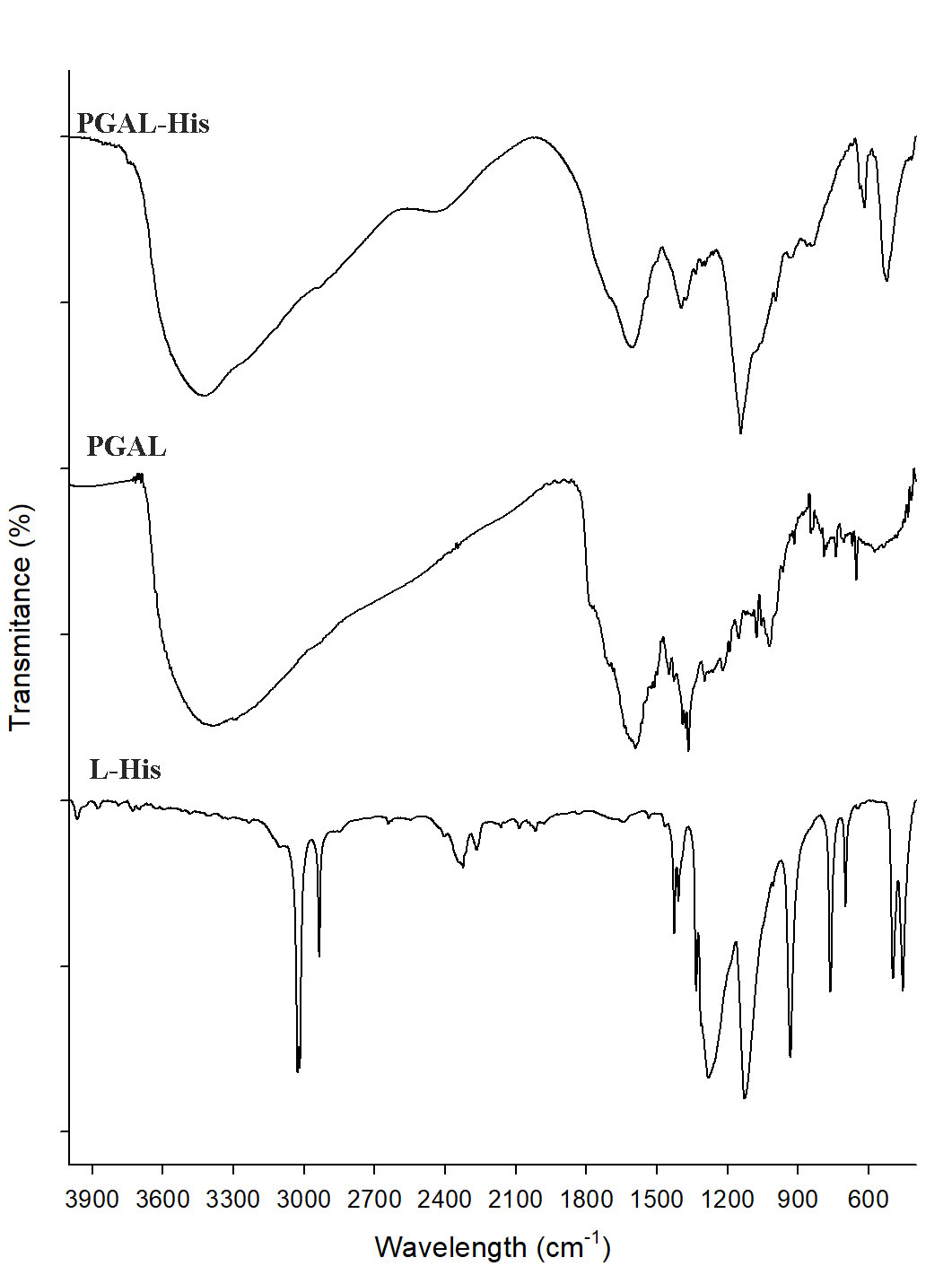


Figure: FT-IR spectra for PGAL-His sample (above); PGAL (middle) and L-Histidine (Below)
